# Characterizing Walking Behaviors in Aged Residential Care Using Accelerometry, With Comparison Across Care Levels, Cognitive Status, and Physical Function: Cross-Sectional Study

**DOI:** 10.2196/53020

**Published:** 2024-06-04

**Authors:** Ríona Mc Ardle, Lynne Taylor, Alana Cavadino, Lynn Rochester, Silvia Del Din, Ngaire Kerse

**Affiliations:** 1 Translational and Clinical Research Institute, Newcastle University, Newcastle Upon Tyne, United Kingdom; 2 National Institute for Health and Care Research Biomedical Research Centre, Newcastle University and the Newcastle Upon Tyne Hospitals National Health Service Foundation Trust, Newcastle Upon Tyne, United Kingdom; 3 School of Population Health, Faculty of Medical and Health Sciences, University of Auckland, Auckland, New Zealand; 4 The Newcastle Upon Tyne Hospitals National Health Institute Foundation Trust, Newcastle Upon Tyne, United Kingdom

**Keywords:** residential aged care facility, cognitive dysfunction, mobility limitation, accelerometry, physical activity, aged residential care

## Abstract

**Background:**

Walking is important for maintaining physical and mental well-being in aged residential care (ARC). Walking behaviors are not well characterized in ARC due to inconsistencies in assessment methods and metrics as well as limited research regarding the impact of care environment, cognition, or physical function on these behaviors. It is recommended that walking behaviors in ARC are assessed using validated digital methods that can capture low volumes of walking activity.

**Objective:**

This study aims to characterize and compare accelerometry-derived walking behaviors in ARC residents across different care levels, cognitive abilities, and physical capacities.

**Methods:**

A total of 306 ARC residents were recruited from the Staying UpRight randomized controlled trial from 3 care levels: rest home (n=164), hospital (n=117), and dementia care (n=25). Participants’ cognitive status was classified as mild (n=87), moderate (n=128), or severe impairment (n=61); physical function was classified as high-moderate (n=74) and low-very low (n=222) using the Montreal Cognitive Assessment and the Short Physical Performance Battery cutoff scores, respectively. To assess walking, participants wore an accelerometer (Axivity AX3; dimensions: 23×32.5×7.6 mm; weight: 11 g; sampling rate: 100 Hz; range: ±8 g; and memory: 512 MB) on their lower back for 7 days. Outcomes included volume (ie, daily time spent walking, steps, and bouts), pattern (ie, mean walking bout duration and alpha), and variability (of bout length) of walking. Analysis of covariance was used to assess differences in walking behaviors between groups categorized by level of care, cognition, or physical function while controlling for age and sex. Tukey honest significant difference tests for multiple comparisons were used to determine where significant differences occurred. The effect sizes of group differences were calculated using Hedges *g* (0.2-0.4: small, 0.5-0.7: medium, and 0.8: large).

**Results:**

Dementia care residents showed greater volumes of walking (*P*<.001; Hedges *g*=1.0-2.0), with longer (*P*<.001; Hedges *g*=0.7-0.8), more variable (*P*=.008 vs hospital; *P*<.001 vs rest home; Hedges *g*=0.6-0.9) bouts compared to other care levels with a lower alpha score (vs hospital: *P*<.001; Hedges *g*=0.9, vs rest home: *P*=.004; Hedges *g*=0.8). Residents with severe cognitive impairment took longer (*P*<.001; Hedges *g*=0.5-0.6), more variable (*P<*.001; Hedges *g*=0.4-0.6) bouts, compared to those with mild and moderate cognitive impairment. Residents with low-very low physical function had lower walking volumes (total walk time and bouts per day: *P*<.001; steps per day: *P*=.005; Hedges *g*=0.4-0.5) and higher variability (*P*=.04; Hedges *g*=0.2) compared to those with high-moderate capacity.

**Conclusions:**

ARC residents across different levels of care, cognition, and physical function demonstrate different walking behaviors. However, ARC residents often present with varying levels of both cognitive and physical abilities, reflecting their complex multimorbid nature, which should be considered in further work. This work has demonstrated the importance of considering a nuanced framework of digital outcomes relating to volume, pattern, and variability of walking behaviors among ARC residents.

## Introduction

Physical mobility, such as walking, is a key predictor of health [1] and is considered a multifaceted experience that interconnects the physical, mental, social, and emotional needs of an individual with their sense of self [2,3]. Loss of physical mobility (eg, reduced volume of walking) is associated with increased safety risks (eg, falls), social withdrawal, and poorer well-being [4,5]. Supporting residents’ physical mobility in aged residential care (ARC) can decelerate the progression of disabilities and dependency [6]. ARC refers to long-term full-time residential care, which provides multiple levels of care depending on an individual’s needs. Other common terms for ARC include assisted living facilities, care homes, and nursing homes. It is recommended that all residents who can ambulate, regardless of cognitive abilities, should increase their activity levels to support their functional independence [7]. Continuous remote digital monitoring of mobility outcomes has been proposed as a method to objectively quantify changes in walking behaviors. This approach will inform the development of interventions aimed at better supporting mobility, which is a key factor in influencing well-being and function in the older population [2].

Accelerometers are the most common method to continuously assess walking behaviors in ARC residents [8], with outcomes relating to volume (eg, steps per day) and intensity (eg, moderate-vigorous physical activity) of walking activities most frequently reported. Based on the current literature, ARC residents primarily participate in low volumes of light-intensity walking and show little variation in their walking behaviors [8]. Based on prevailing gaps in the literature, current recommendations for the assessment of walking behaviors in ARC include the use of validated digital methods that can capture very low volumes of activity, using low cutoff thresholds (eg, any walking activity ≥3 steps), and derive standardized outcomes relating to volume (ie, the amount or duration of walking activity), pattern (ie, the distribution of walking activity across a time period), and variability (ie, changes in walking activities—either within-person or group activities—and over time) of walking behaviors [8,9].

Using this nuanced framework, we can consider how different attributes impact discrete walking behaviors. For example, we previously found that better physical function was associated with higher walking volumes in ARC residents in intermediate (ie, rest homes) and high-level (ie, hospitals) care, while surprisingly, moderate dementia, mild depression, and pain had no effect on walking volumes [10]. In contrast, people with mild cognitive impairment in the community show no differences in walking volumes compared to people who undergo normal aging, but they do demonstrate different patterns and greater variability in their walking behaviors [9]. By looking beyond the volume of walking activities to pattern and variability, we may garner information about people’s routines and the time they spend indoors and outdoors (based on walking bout lengths) [8,9,11-14]; we can then examine the impact of cognitive and physical impairments on these behaviors [14]. This information can contribute toward the development of more holistic interventions to support mobility in ARC.

Notably, ARC residents are a complex multimorbid population with significant variation in cognitive and physical function, often reflected in the level of care provided. These heterogeneities are not reflected in the literature regarding walking behaviors, highlighting a clear gap [8,15,16]. For example, Mc Ardle et al’s [8] review on the quantification of ambulatory activities in ARC reported that 26% of studies excluded people with cognitive impairment and only 17% explicitly characterized walking activities in people with cognitive impairment, despite a 65%-70% prevalence of cognitive impairment in ARC residents [17,18]. Additionally, no studies compared different levels of care. As such, Mc Ardle et al [8] recommended that we must characterize and compare the volumes, patterns, and variability of walking behaviors in ARC residents across different care levels, with different cognitive and physical abilities. By characterizing walking behaviors in a representative group of ambulatory ARC residents, we can gain a better understanding of physical mobility in ARC, which will inform future interventions and policies to promote walking activities and support mobility and function in ARC residents.

To address the highlighted gaps and recommendations, the primary aim of this study was to digitally characterize and compare walking behaviors across different levels of ARC using a validated and standardized framework, encompassing volume, pattern, and variability of walking. Secondary aims of this study were to characterize and compare walking behaviors in ARC residents according to their cognitive status and physical function.

## Methods

### Participants

Residents from 24 ARC facilities in New Zealand were recruited as part of the Staying UpRight randomized controlled trial (RCT), which evaluated an exercise intervention to reduce fall risk [19]. Only baseline data are included in this study. Participants were included if they were aged ≥65 years and mobile (ie, able to walk and transfer independently or with supervisory assistance). Participants were receiving one of the following levels of care: hospital-level care (24-hour care by, or under the supervision of, a nurse), rest home–level care (24-hour health-related care but not nursing care), or dementia-level care (rest-home level care in a secure environment to minimize the risks associated with dementia).

We excluded residents in psychogeriatric, respite, or palliative care; residents unable to undertake the assessment or the exercise intervention in the main RCT because they were acutely unwell (eg, gastroenteritis), or immobile (ie, unable to mobilize without 2-person assistance or bed bound) were also excluded.

### Ethical Considerations

Participants who were able to give informed written consent did so before enrollment, and the facility clinical lead provided written consent for residents unable to provide their own informed consent because of cognitive impairment. The study was conducted according to the guidelines of the Declaration of Helsinki. Ethics approval was provided by the New Zealand Health and Disability Ethics Committee on October 31, 2018 (NZHDEC 18/NTB/151).

### Clinical and Cognitive Outcomes

Demographic information for ARC residents included the following: age, sex, and years spent in the ARC facility. Physical function was measured using Short Physical Performance Battery (SPPB) [20] and the Timed Up and Go test [21]. Cognitive ability was assessed using the Montreal Cognitive Assessment (MoCA) [22].

### Assessment of Walking Behaviors

ARC residents were asked to wear a small body-worn accelerometer (Axivity AX3; dimensions: 23×32.5×7.6 mm; weight: 11 g; sampling rate: 100 Hz; range: ±8 g; and memory: 512 MB) on the fifth lumber vertebra on the lower back. The accelerometer was affixed onto the skin using a double-sided hydrogel adhesive and a hypoallergenic plaster (Hypafix BSN Medical Limited). This particular protocol has been found to be feasible for multisite studies [23] in different aging cohorts [11,13,24]. Of particular note, algorithms used in this study for walking bout detection have been validated in ARC residents, with high accuracy for start and end time [25].

Participants were asked to wear the accelerometer continuously for 7 days, including in the shower and to bed. Once the assessment was complete, data were downloaded to a computer and processed via a validated analytical pipeline in MATLAB.

### Data Processing and Walking Behavior Outcomes

Signals from the accelerometer were transformed to a horizontal-vertical co-ordinate system. Walking bouts were identified by filtering raw acceleration data using a second-order low-pass Butterworth two-pass digital filter, with a cutoff frequency of 17 Hz, and by applying selective thresholds on the vector magnitude and standard deviations of triaxial acceleration signals [11,23,26,27]. Once walking bouts were identified, for detecting steps, raw acceleration signals were filtered with low-pass, fourth-order Butterworth filter with cutoff frequency of 20 Hz. A Gaussian continuous wavelet transform of vertical acceleration was then applied to identify initial and final contacts, allowing the identification of steps. For each walking bout, total steps per bout and bout length were calculated. Sleep, lying, and sitting data were excluded based on the thresholds applied on the magnitude and standard of the accelerometry signal used to identify walking (eg, vertical acceleration, in a vertical position, needs to be −1 g and acceleration magnitude or standard needs to exceed these thresholds to be classified as walking). For sleep, the magnitude and standard of acceleration would be lower and the vertical acceleration would not be −1 g, so the position (orientation) excludes sleep, lying, or sitting.

A framework of walking behaviors was derived to remain consistent with previous literature [11,12,23,26], including volume, pattern, and variability of walking. Volume characteristics included total minutes spent walking as well as steps and bouts per day. Pattern characteristics included mean bout duration and alpha, which is derived by logarithmic transformation of bout density and length and is based on shape and power-law distribution [28,29]; alpha refers to the ratio of short to long walking bouts, which are scaled relative to an individual’s shortest walking bout. A high alpha score indicates that an individual’s total walking time is composed of proportionally shorter walking bouts compared to long walking bouts. Variability (S_2_) refers to the variability of bout duration between walking bouts, estimating how much an individual’s bout duration changes over the time period of data collection, and it was estimated using the maximum likelihood technique (previously described by Mc Ardle et al and Del Din [9,13]. The proportion of walking bouts taken in very short (<10 s), short (10-30 s), medium (30-60 s), and prolonged (>60 s) walks were calculated. These walking bout thresholds have been used commonly in other studies of a similar nature and provide contextual information regarding how walking takes place [13,30,31].

### Considerations for Inclusion of Data

Given that most habitual walking takes place in <10-second bouts [13,32,33], we applied a minimum bout duration of 3 consecutive steps, and any period of rest that was ≥2.5 seconds was considered resting time [32]. Additionally, we included participants if they had ≥2 days of continuous walking activity data collected, as this is the minimum number of days required to reliably quantify our primary outcomes (ie, the volume of walking) across different care levels, based on Buckley et al [27].

### Data Analysis

For demographic variables, chi-square tests and Fisher exact test were used to determine differences between groups for nominal variables, while one-way ANOVA was used to determine between-group differences for continuous variables; post hoc Tukey honest significant difference (HSD) tests determined where the differences lay.

Prior to statistical analysis relating to our primary and secondary aims, walking activity data were inspected visually using box plots, and outliers were identified. Separate analyses of covariance were used to assess differences in walking behaviors between groups categorized by level of care, cognition, or physical function while controlling for age and sex. Tukey HSD tests for multiple comparisons were used to determine where significant differences occurred. Sensitivity analysis was conducted by removing outliers more than 1.5 times above the third quartile or below the first quartile and by conducting the analysis of covariance and subsequent post hoc tests for each discrete grouping separately (eg, level of care, cognition, or physical function).

The effect size of group differences was calculated using the Hedges *g* formula to account for disparities between groups’ sample sizes [34]. Effect sizes are interpreted as follows: 0.2-0.4: small, 0.5-0.7: medium, and ≥0.8: large. Assumptions were evaluated (eg, normality of residuals) for all models, and statistical significance was defined as a *P*<.05.

Cognitive levels were assessed and categorized using MoCA cutoff scores, as follows: cognitively intact (MoCA ≥26), mild cognitive impairment (MoCA 18-25), moderate cognitive impairment (MoCA 10-17), and severe cognitive impairment (MoCA <10) [35]. Cognitively intact participants were excluded from the cognitive impairment severity analysis due to the small sample size but retained for illustrative purposes in Figures. Physical function levels were assessed and categorized using the SPPB cutoff scores, as follows: high-moderate function (SPPB 12-7) or low-very low function (SPPB <7) [36].

## Results

### Demographic Information

**Figure 1.**
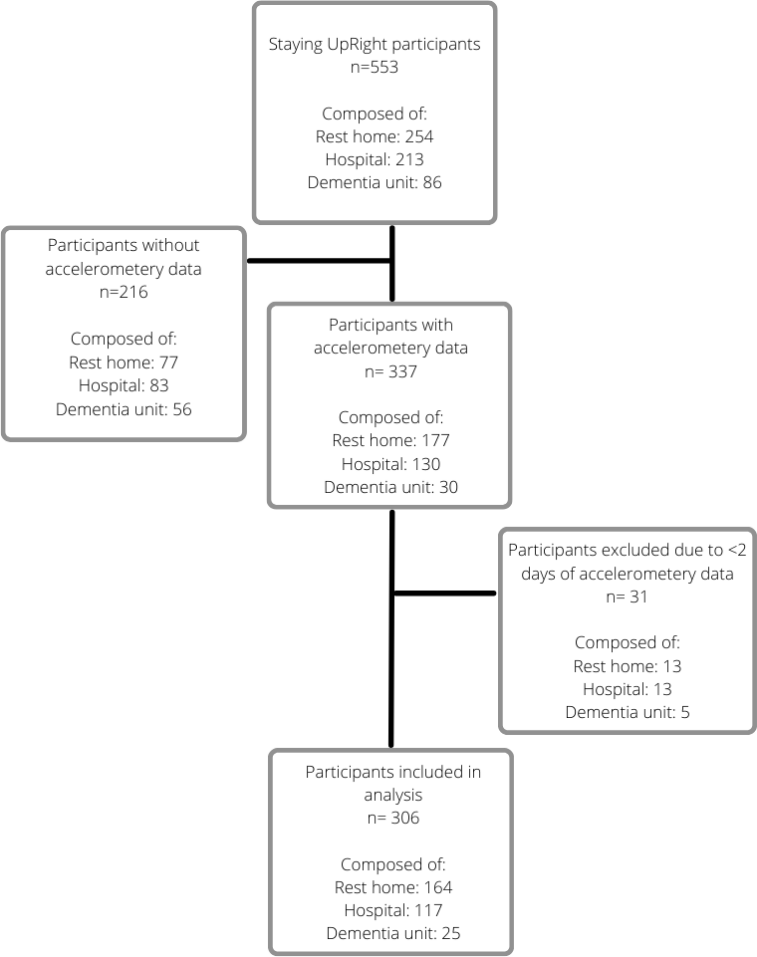
Flowchart for inclusion of participants in this analysis.

A total of 306 ARC residents were included in this analysis and were primarily grouped according to their level of care (Table 1). Figure 1 outlines reasons for exclusion and inclusion of participants for this secondary analysis from the Staying UpRight RCT. Hospital-level care residents had lower physical function compared to rest home–level care residents (*P*=.01) and took a longer time to complete the Timed Up and Go test compared to rest home–level (*P*<.001) and dementia-level (*P*=.03) residents. MoCA scores were significantly lower in dementia-level residents compared to rest home–level and hospital-level care residents (*P*<.001).

**Table 1.** Demographic information for participants categorized by levels of care. Italicized *P* values indicate significance.

Characteristics		Hospital (n=117)	Rest home (n=164)	Dementia care (n=25)	Overall *P* value^a^
Age (years; n=306), mean (SD)		84 (7)	84 (7)	81 (8)	.20
**Sex (n=306), n (%)**					.90
	Female	70 (60)	101 (62)	16 (64)	
	Male	47 (40)	63 (38)	9 (36)	
Years in facility^b^ (n=304), mean (SD)		0.4 (0.2)	0.4 (0.2)	0.4 (0.1)	.60
SPPB^c^ score (0-12; n=296), mean (SD)		4.3 (2.6)^d^	5.2 (2.6)^d^	4.1 (2.3)	*.008*
**Physical function level**^e^ **(n=296), n (%)**					—^f^
	High physical function (SPPB 10-12)	5 (4.3)	9 (5.6)	0 (0)	
	Moderate physical function (SPPB 7-9)	19 (17)	38 (24)	3 (15)	
	Low physical function (SPPB 4-6)	37 (32)	70 (43)	8 (40)	
	Very low physical function (SPPB <4)	54 (47)	44 (27)	9 (45)	
	Unknown^g^	2	3	5	
TUG^h^ (s; n=289), mean (SD)		37 (22)^d^^,^^i^	27 (15)^d^	25 (18)^i^	*<.001*
MoCA^j^ score (0-30; n=285), mean (SD)		15 (6)^i^	15 (6)^k^	4 (6)^i^^,^^k^	*<.001*
**Cognitive level**^e^ **(n=285), n (%)**					—
	Cognitively intact (MoCA ≥26)	5 (4.6)	4 (2.5)	0 (0)	
	Mild cognitive impairment (MoCA 18-25)	28 (26)	58 (36)	1 (5.6)	
	Moderate cognitive impairment (MoCA 10-17)	60 (56)	66 (42)	2 (11)	
	Severe cognitive impairment (MoCA <10)	15 (14)	31 (19)	15 (83)	
	Not tested^l^	9	5	7	
Days wearing the activity monitor (n=306), mean (SD)		6.5 (1)	6.4 (1)	6.3 (1)	.60

a One-way ANOVA, Pearson chi-square test, and Fisher exact test.

b For years in facility, 1 partcipant’s data were missing from both the “hospital” and “rest home” groups.

c SPPB: Short Physical Performance Battery (2 participants in the “hospital” group, 3 participants in the “rest home” group, and 5 participants in the “dementia care” group were not tested for SPPB).

d Hospital vs rest home.

e Descriptive variable only (no statistical testing performed).

f Not applicable.

g “Unknown” indicates participant data missing in each group, so percentages are not applicable.

h TUG: Timed Up and Go (6 participants in the “hospital” group, 3 participants in the “rest home” group, and 8 participants in the “dementia care” group were not tested).

i Hospital vs dementia care.

j MoCA: Montreal Cognitive Assessment (9 participants in the “hospital” group, 5 participants in the “rest home” group, and 7 participants in the “dementia care” group were not tested).

k Rest home vs dementia care.

l Indicates the number of participants not tested in each group, so percentages are not applicable.

### Walking Behaviors Across Care Levels

Dementia care residents demonstrated higher volumes of walking, with longer, more variable bout durations and lower alpha scores compared to residents in both rest homes and hospitals (moderate to large effect sizes). Residents in rest homes also showed higher volumes of walking compared to those in hospitals (small effect sizes). Table 2 provides further details; Multimedia Appendix 1 provides *P* values for post hoc tests and details regarding effect sizes. Notably, sensitivity analysis indicated that variability of walking bout length did not differ between groups following the removal of outliers (*P=*.16).

**Table 2.** Characterization of walking behaviors, categorized by level of care (N=306).

Characteristics		Hospital (n=117)	Rest home (n=164)	Dementia care (n=25)	Overall *P* value^a^
Walk time per day (min), mean (SD)		58 (37)^b,c^	74 (39)^b^^,^^d^	137 (59)^c^^,d^	<.001
Steps per day, mean (SD)		4138 (2766)^b^^,c^	5216 (2925)^b^^,d^	10,886 (5453)^c^^,^^d^	<.001
Bouts per day, mean (SD)		256 (165)^b^^,^^c^	321 (160)^b^^,d^	496 (238)^c^^,^^d^	<.001
Mean bout duration (s), mean (SD)		13.9 (3.6)^c^	14.1 (3.3)^d^	20.1 (20.4)^c^^,^^d^	<.001
Variability, mean (SD)		0.81 (0.11)^c^	0.80 (0.09)^d^	0.89 (0.18)^c^^,^^d^	.002
Alpha score, mean (SD)		1.68 (0.08)^c^	1.67 (0.07)^d^	1.61 (0.09)^c^^,^^d^	<.001
**Distribution of walking bouts by discrete walking bout length (%), mean (SD)**					
	<10-second bouts	65 (8)^c^	64 (8)^d^	56 (10)^c^^,^^d^	<.001
	10- to 30-second bouts	25.9 (5.5)^c^	27.6 (5.5)^d^	30.8 (5.8)^c^^,^^d^	<.001
	30- to 60-second bouts	5.81 (3.05)^c^	5.51 (2.42)^d^	8.64 (3.64)^c^^,^^d^	<.001
	>60-second bouts	3.20 (2.13)^c^	3.15 (2.06)^d^	4.82 (4.92)^c^^,^^d^	.01

a One-way ANOVA, controlling for age and sex.

b Hospital vs rest home.

c Hospital vs dementia care.

d Rest home vs dementia care.

Additionally, dementia care residents spent a significantly lower percentage of their walking bouts in very short bouts (eg, <10 s) and a greater percentage in short, medium, and prolonged walking bouts compared to residents in other care levels (moderate to large effect sizes; Table 2).

### Walking Behaviors Across Cognitive Impairment Severities

**Figure 2.**
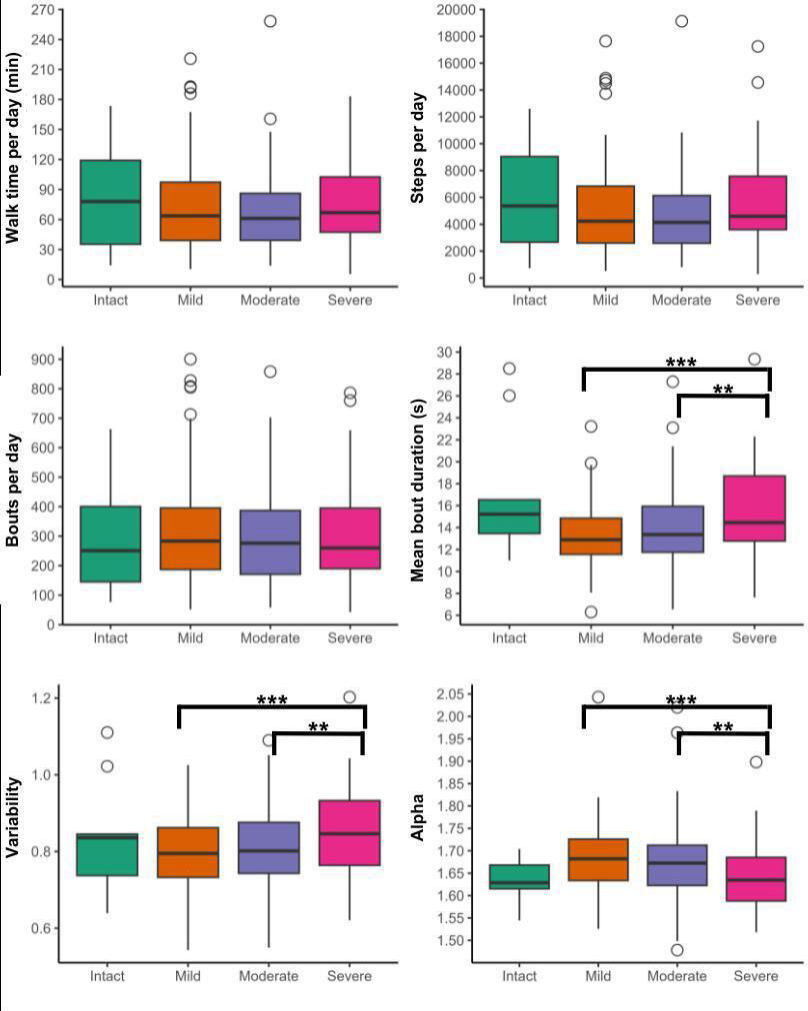
Volume, pattern, and variability of walking behaviors across cognitive groups. ***P≤.001; **P≤.01.

There were no significant differences between cognitive groups for any volume characteristics (Figure 2 and Multimedia Appendix 2). People with severe cognitive impairment took longer, more variable walking bouts with a lower alpha score compared to those with mild (moderate to large effect sizes) and moderate cognitive impairment (small to moderate effect sizes). Figure 2 and Multimedia Appendix 2 provide further details.

### Walking Behaviors Across Physical Function Levels

**Figure 3.**
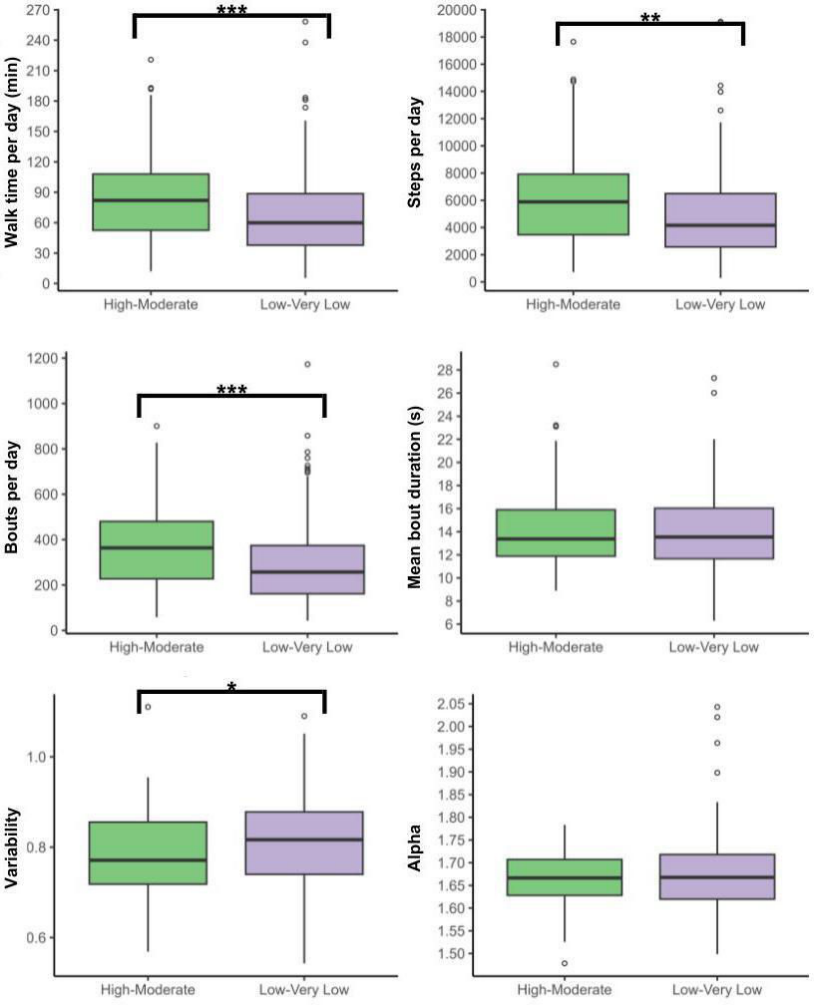
Volume, pattern, and variability of walking behaviors across physical function groups. ***P≤.001; **P≤.01.

ARC residents with high-moderate physical function spent more time walking and took more steps and bouts per day, with less variability for bout length, compared to those with low-very low physical function (Figure 3 and Multimedia Appendix 3 present further details).

## Discussion

### Principal Findings

This is the first study to describe the volume, pattern, and variability of walking behaviors in ARC, captured by an accelerometer, with consideration of different care levels, cognitive abilities, and physical function, reflecting the typical population of residents. Key findings highlight that dementia care residents have significantly higher volumes of walking, take longer and more variable walking bouts on average, and spend proportionately more of their walking time in prolonged bouts of walking compared to rest home and hospital levels of care. Although the volume of walking is similar across different levels of cognitive impairment severity, people with more severe cognitive impairment show different patterns (eg, longer walking bouts) and greater variability compared to those who are less cognitively impaired. In contrast, people with lower physical function have significantly lower volumes of walking and higher variability of walking bout lengths but do not differ in terms of the pattern of this activity. ARC residents are a complex multimorbid population who often present with varying levels of both cognitive and physical abilities, and these nuances should be considered in further research aiming to improve mobility and reduce fall risk.

### Walking Behaviors Across Different Care Environments

This is the first study to show that people living in a dementia unit participate in higher volumes of walking, with different patterns (ie, longer walking bouts) and greater variability compared to other ARC environments, with medium to large effect sizes. However, differences between groups for the variability of walking bout length disappeared following the removal of significant outliers, therefore, results should be interpreted with caution. As physical function scores are comparable between the dementia care and rest home residents (Table 1), the differences in walking behaviors may illustrate a behavioral component of dementia (eg, wandering—a dementia-related locomotor behavior involving frequent and repetitive movements, such as pacing). Objective remote monitoring of wandering behaviors using digital methods has previously been proposed to detect and monitor wandering behaviors [37,38]. We propose that variability of walking bout lengths should be considered in future research in this area, as it may reflect wandering behaviors[39]; clinical validation is required to investigate this hypothesis.

Contrary to our findings, baseline results from one previous RCT reported very low volumes of walking activity in dementia care units, showing <400 steps in one 24-hour period assessed via an activity armband [40]. However, these studies are difficult to compare due to differences in device location (ie, lower back compared to arm) and data collection periods. Moyle et al [40] noted that these activity armbands were unreliable and resulted in large amounts of missing data. Additionally, high volumes of walking activity reported here (eg, >4000 steps per day) are likely due to our low cutoff thresholds for defining walking activity, as most walking takes place in very short walking bouts in this population. The cutoff threshold applied to characterize walking behaviors can make significant differences in the volume of walking captured—with differences ranging from 2000 to 10,000 steps in previous literature [30]. This study has expanded beyond simple volume metrics and highlighted the importance of selecting validated and sensitive digital methods when assessing walking behaviors in this population [8].

### Walking Behavior Across Cognitive Impairment Severity and Physical Capacities

This is the first study to demonstrate that people with severe cognitive impairment have similar volumes but significantly different patterns (ie, longer bouts) and greater variability of walking compared to less cognitively impaired groups, suggesting that although cognition does not influence the amount of activity, it may change the way this activity is carried out. This finding is supported by and extends our previous work, which excluded dementia care residents and highlighted that while worse physical function is associated with lower volumes of walking in ARC, cognitive impairment showed no effect on walking volume [10]. Perhaps this indicates that pattern and variability of walking behaviors are cognitively mediated outcomes and may be useful to monitor in ARC as a proxy for cognitive decline. For example, in line with our results from the dementia care unit, the literature indicates that people with severe dementia are more likely to wander [41] and we propose that this is reflected in the pattern and variability of walking. Longer walking bouts and higher variability of bout length are considered positive outcomes in cognitively healthy individuals, indicative of dynamic and varied routines [11], but perhaps higher variability in tandem with significant cognitive impairment is more reflective of repetitive lapping behaviors (ie, wandering). Clinical validation is required to address this speculation. Differences in pattern of walking behaviors have previously been reported between community-dwellers with mild cognitive impairment and normal aging [42], supporting the hypothesis that cognitive decline may influence these behaviors.

In contrast, people with worse physical function have significantly lower volumes of walking but show no differences in pattern or variability compared to those with better physical function. The association between higher walking volumes and better physical function confirms the findings of previous studies [43,44]. However, the cross-sectional design of this study precludes commentary on the direction of causality. Although from our results, we cannot determine if encouraging walking as part of a resident’s daily activities can result in clinically meaningful improvements in function, previous research demonstrated that function-focused care (ie, increasing routine activities) leads to increased activity volumes and improved functional outcomes in ARC residents with moderate functional dependency [44] but not in dementia residents with severe functional dependency [45]. However, pattern and variability of walking are considered to reflect daily routines, and the effects of function-focused care may be more readily observed in these outcomes rather than in volume, especially in individuals with severe cognitive impairment. Additionally, marginal increases in the duration and variability of walking bouts may lead to significant improvements in function [28] and should be considered in very frail residents. These hypotheses could be considered in future intervention studies, with consideration for the multimorbid nature and varying levels of both cognitive and physical issues inherent in ARC residents.

### Strengths and Limitations

Strengths of this study were the large sample, drawn from multiple facilities, distributed across 3 levels of care and encompassing a broad spectrum of cognitive and physical capacities. This is particularly notable, as there can be significant difficulties in collecting data using wearable technology from people with dementia in ARC facilities [29]. We used a technically appropriate digital method to collect low volumes of walking data¸ meeting the recommendations from Mc Ardle et al [8]. Additionally, we used a standardized framework to characterize walking behaviors, making our findings comparable to multiple other cohorts and enhancing our understanding of walking behaviors across the spectrum of care and cognition [11,12]. We addressed the reliability of our primary outcomes based on previous empirical evidence [27].

Our study has several limitations. Residents were only included if they could ambulate, and residents who could not complete the MoCA or SPPB were not included in our secondary analysis; therefore, we may have reduced representation of different levels of cognitive and physical capacities. Although this is only the second study to specifically characterize walking activity in a dementia care unit [8], it should be noted that our sample size for this group was low and likely to have limited statistical power; therefore, statistical analysis was exploratory and results should be considered with that in mind. This is a cross-sectional study; therefore, assessing changes in walking activity over time or establishing causality of influences on walking activity is not possible; in the future, a longitudinal study may offer valuable insights into predictors of walking behaviors in ARC. Although we adjusted for multiple corrections within statistical models (ie, Tukey HSD tests), we did not adjust for multiple comparisons for multiple outcomes, and there may be a risk of type I error. We also included participants with ≥2 days of walking activity data, as this is the number of days required to obtain reliable volume outcomes (ie, our primary outcome) in ARC [27]; however, our secondary outcomes of pattern and variability require 2-5 days of data to ensure reliability, pending on the discrete variable, and thus, results should be interpreted with caution. Although commonly assessed in ARC [8], we did not include outcomes relating to the intensity of walking activity, as this has been suggested to be inappropriate to characterize in this population, given that ARC residents primarily engage only in light-intensity activities [8]. Additionally, the ARC facilities included in this study reflect a New Zealand context, and findings may be different in other countries due to alternative organizational features and policies [46]. We recognize that apart from resident-related factors of physical function and cognition, walking activity may be influenced by the physical and organizational environment [46]—aspects that were not measured in our study. As previously noted, ARC residents may have varying levels of both cognitive and physical impairments, and the combined spectrum should be considered in future research. Finally, digital outcomes beyond those described in this analysis can provide important clinical information about ARC residents and should be considered in future research. For example, sleep disturbances can be measured using actigraphy. Sleep disturbances are common in people living in ARC and are associated with neuropsychiatric symptoms and prescription of psychotropic drugs, which can enhance fall risks and greater staff distress [47]. Although it is beyond the scope of this study, further research may consider using qualitative approaches to complement current findings and the wider literature [8], which would allow us to garner rich insights from ARC residents regarding which digital outcomes relate to their lived experiences and are meaningful to assess.

### Conclusions

This is the first study to show the influence of care environment, cognitive status, and physical function on walking behaviors in ARC residents. Our results indicate that cognitive and physical abilities may discretely impact the volumes, pattern, and variability of walking. This work has addressed a significant gap in the literature and has generated new hypotheses regarding which digitally derived walking outcomes are meaningful to assess in ARC residents.

## Supplementary material

10.2196/53020 Multimedia Appendix 1 Detailed description of between-group analysis results for different care levels.

10.2196/53020 Multimedia Appendix 2 Walking behaviors categorized by cognitive impairment severity.

10.2196/53020 Multimedia Appendix 3 Walking behaviors categorized by physical function.

## Abbreviations

ARC: aged residential care
HSD: honest significant difference
MoCA: Montreal Cognitive Assessment
RCT: randomized controlled trial
SPPB: Short Physical Performance Battery
