# Supplementary material for: Characterizing Walking Behaviors in Aged Residential Care Using Accelerometry, With Comparison Across Care Levels, Cognitive Status, and Physical Function: Cross-Sectional Study
Source: JMIR Aging. 2024 Jun 4;7:e53020. doi: 10.2196/53020 (PMC11185191; doi:10.2196/53020)
Supplement: Multimedia Appendix 1 [file aging-v7-e53020-s001.docx]

**Multimedia Appendix 1: Detailed description of between-group analysis results for different care levels.**

## Care Levels

### Walking behaviours across care levels

When controlling for age and sex, residents in the dementia unit spent more time walking (*p<.001*, Hedges’ g for hospital = 1.9, for rest home = 1.5), took more steps (*p<.001*, Hedges’ g for hospital = 2.0, for rest home = 1.7) and bouts (*p<.001*, Hedges’ g for hospital = 1.3, for rest home = 1.0), had longer (*p<.001*, Hedges’ g for hospital = 0.7, for rest home =0.8) and more variable bout durations (vs hospital, p=.008, Hedges’ g = 0.6; vs rest home, p=.001, Hedges’ g = 0.9) and demonstrated a lower alpha score (vs hospital, *p<.001*, Hedges’ g = 0.9; vs rest home, *p=.004*, Hedges’ g = 0.8) compared to both hospital and rest home residents (see Table 1). Rest home residents also spent more time walking (*p=.003*, Hedges’ g = 0.4), took more steps (*p=.01*; Hedges’ g = 0.4) and bouts (*p=.005*, Hedges’ g = 0.4) compared to hospital residents.

Additionally, residents in the dementia unit took a significantly lower percentage of their walking bouts in very short bouts (*p<.001*; vs hospital, Hedges’ g=1.1; vs rest home, Hedges’g=1.0), and a greater percentage of their walking bouts in short (vs hospital*, p<.001*, Hedges’ g=0.9; vs rest home, *p=.02*, Hedges’ g=0.6), medium (*p<.001*; vs hospital, Hedges’ g=0.9; vs rest home, Hedges’g=1.2) and prolonged walking bouts (vs hospital, *p=.01*, Hedges’ g=0.6; vs rest home, *p=.007*, Hedges’ g=0.6) compared to hospital and rest home residents (see Table 2).
