# Supplementary material for: Characterizing Walking Behaviors in Aged Residential Care Using Accelerometry, With Comparison Across Care Levels, Cognitive Status, and Physical Function: Cross-Sectional Study
Source: JMIR Aging. 2024 Jun 4;7:e53020. doi: 10.2196/53020 (PMC11185191; doi:10.2196/53020)
Supplement: Multimedia Appendix 2 [file aging-v7-e53020-s002.docx]

**Appendix 2: Walking behaviours categorised by cognitive impairment severity**

|  | | **N** | | **Mild cognitive impairment**, N = 87^1^ | | **Moderate cognitive impairment**, N = 128^1^ | | **Severe cognitive impairment**, N = 61^1^ | | | **Overall**  **p-value**^2^ | | | |
| --- | --- | --- | --- | --- | --- | --- | --- | --- | --- | --- | --- | --- | --- | --- |
| **Walk time per day (mins)** | 276 | | 74(47) | | 66(36) | | 75(41) | | | .30 | | | |  |
| **Steps per day** | 276 | | 5,218(3,623) | | 4,607(2,604) | | 5,614(3,324) | | | .10 | | | |  |
| **Bouts Per Day** | 276 | | 327(196) | | 289(149) | | 301(171) | | | .30 | | | |  |
| **Mean Bout Durations (secs)** | 276 | | 13.4(3.0)^a^ | | 13.9(3.1)^b^ | | 15.5(4.1)^a,b^ | | | <.001 | | | |  |
| **Variability** | 276 | | 0.79(0.10)^a^ | | 0.81(0.09)^b^ | | 0.85(0.11) ^a,b^ | | | .002 | | | |  |
| **Alpha** | 276 | | 1.69(0.07)^a^ | | 1.67(0.07)^b^ | | 1.64(0.07) ^a,b^ | | | <.001 | | | |  |
| ^1^Mean(SD) | | | | | | | | |  | | |  |  |  |
| ^2^One-way ANOVA, controlling for age and sex | | | | | | | | |  | | |  |  |  |

## Cognitive levels

## Walking behaviours across cognitive levels

There were no significant differences between cognitive groups for any volume characteristics (*p>.05*; see Figure 1). When controlling for age and sex, people with severe cognitive impairment showed longer (*p<.001*, Hedges’ g = 0.6, 15.5±4.1 vs 13.4±3 seconds), more variable walking bouts(*p<.001*; Hedges g= 0.6, .85±.11 vs .79±1) with a lower alpha score (*p<.001*; Hedges g=0.7,1.64 ±.07 vs 1.69±.07) compared to those with mild cognitive impairment, and also demonstrated longer (*p=.007*, Hedges’ g = 0.5, 15.5±4.1 vs 13.9±3.1 seconds), more variable walking bouts (*p=.01*, (p<.007; Hedges g= 0.4, 85±.11 vs .81±.09) with a lower alpha score (*p=.01*; Hedges g=0.4,1.64 ±.07 vs 1.67±.07) compared to those with moderate cognitive impairment.
