# Supplementary material for: Characterizing Walking Behaviors in Aged Residential Care Using Accelerometry, With Comparison Across Care Levels, Cognitive Status, and Physical Function: Cross-Sectional Study
Source: JMIR Aging. 2024 Jun 4;7:e53020. doi: 10.2196/53020 (PMC11185191; doi:10.2196/53020)
Supplement: Multimedia Appendix 3 [file aging-v7-e53020-s003.docx]

Appendix 3: **Walking behaviours categorised by physical function**

| **Characteristic** | **N** | **High-Moderate Physical Function**, N = 74^1^ | **Low-Very Low Physical Function**, N = 222^1^ | **p-value**^2^ |
| --- | --- | --- | --- | --- |
| **Walk time per day (mins)** | 296 | 87(48) | 67(40) | <.001 |
| **Steps per day** | 296 | 6,143(3,680) | 4,796(3,042) | .005 |
| **Bouts Per Day** | 296 | 375(196) | 292(172) | <.001 |
| **Mean Bout Duration (secs)** | 296 | 14.1(3.5) | 14.0(3.4) | .90 |
| **Variability** | 296 | 0.79(0.10) | 0.81(0.10) | .04 |
| **Alpha** | 296 | 1.66(0.06) | 1.68(0.08) | .20 |
| ^1^Mean(SD) | | | | |
| ^2^One-way ANOVA | | | | |

Residents with high-moderate physical function spent more time walking (*p<.001;* Hedges g = 0.5), and took more steps (*p=.005*; Hedges g = 0.4) and bouts per day *(p<.001*; Hedges g = 0.5), with less variability for bout length (*p=.04*; Hedges g = 0.2) compared to those with low-very low physical function.
